# Managing Pregnancy in Nazi Concentration Camps: The Role of Two Jewish Doctors

**DOI:** 10.5041/RMMJ.10347

**Published:** 2018-07-30

**Authors:** George M. Weisz, Konrad Kwiet

**Affiliations:** 1 School of Humanities, University of New England, Armidale, New South Wales, Australia; 2 Emeritus Professor, Sydney University, Sydney, Australia; 3 Resident Historian, Sydney Jewish Museum, Sydney, Australia

**Keywords:** Concentration camp births, persecuted physicians, survival

## Abstract

Despite daunting circumstances, history is full of stories of men and women incarcerated by the Nazis, who risked their lives to save others. In some cases, the moral dilemma faced by these people presented an unquestionable challenge—particularly for those in the medical profession who had taken an oath to save life. This paper presents the dramatic stories of Dr. Gisella Perl and Dr. Erno Vadasz. Although their choices were markedly different, their goals were the same—to save as many lives as possible.

## INTRODUCTION

Unprecedented horrific crimes against humanity were committed during World War II. In Continental Europe the “Final Solution of the Jewish Question” embarked upon by the Nazis led to the slaughter of six million Jewish men, women, and children. The murder of more than an estimated one-and-a-half million children was considered justifiable by the Nazis, to prevent “… the revengers, in the form of children, to grow up and face our sons and grandsons.”^1^ Even if able to work, pregnant women went to the gas chambers upon arrival. If they managed to hide their pregnancies, their newborn babies were killed either by lethal injection or by drowning.^2^,^3^

The only way the mother could escape the death sentence was by undergoing a secret abortion or by suffocating the newborn, to prevent detection of the birth as anything other than a “still birth,” and to protect all involved in saving the mother’s life.

The total number of Jewish babies killed in the concentration camps is unknown, but the numbers must have been high, based on the number of female prisoners. Only at the end of the war, when hundreds of non-Jewish women with babies were released, was the extent of this slaughter understood—in comparison only a few dozen Jewish mothers were released alive with their newborn children.^4^

This paper explores the very different choices of two physicians involved in either saving the mothers or saving both the mothers with their babies. Core to their actions was a commitment to hold to their medical values. Faced with a lose–lose situation, their oaths as physicians to “do no harm” forced them instead to act in the interests of what would do the *least* harm—to their patients and to themselves. The ethical aspects of this topic have been discussed previously by Chelouche.^5^

## DR. GISELLA PERL—PROTECTOR OF MOTHERS

**Figure 1:**
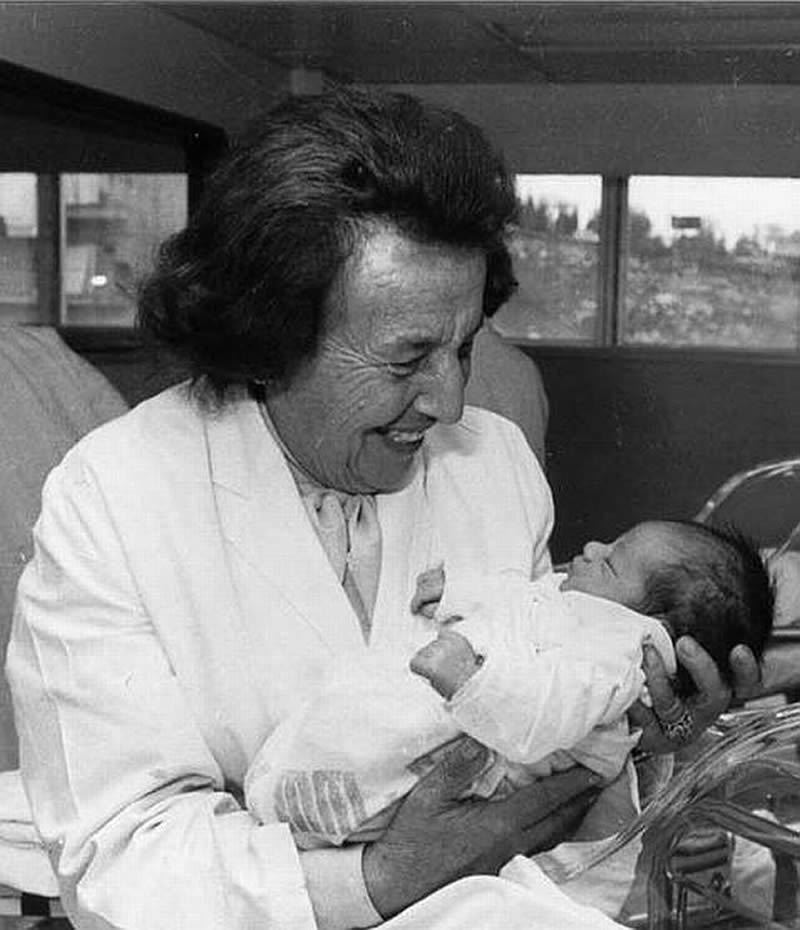
Dr. Gisella Perl after World War II. Credit: Public Domain

**Figure 2:**
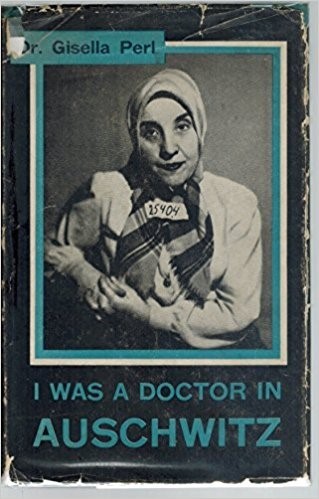
Cover of Dr. Perl’s Book, Published after World War II

The first post-war medical testimonies were given by physicians in the Auschwitz–Birkenau concentration camp. Key testimonies were given by Gisella Perl (Figure 1), Olga Lengyel, Lucie Aldsberger, and Miklos Nyiszly.^6^–^8^ Dr. Perl went on to write a book about her experience (Figure 2).^2^

### Background and Medical Work

Dr. Gisella Perl was born in 1907 to an orthodox Jewish family in Sighet, a small town in Transylvania (Romania). She was a precocious child who became the first Jewish girl in town to finish high school. She also became the first Jewish woman admitted to University Medical School in Kolosvar. After graduating with merit, Perl established a family and gave birth to a son and a daughter. She became a successful and well-known gynecologist in Sighet, conducting a busy medical practice until 1944.

### A Gynecologist in Auschwitz

Following the Nazi invasion of northern Romania in 1944, Perl was deported with her family to Auschwitz. Her daughter remained behind with a “Righteous Gentile” family. The family arrived at the camp after a four-day train ride and were subsequently separated from each other; she would never see them again.

Perl was initially tasked with encouraging blood “donations” from the Jewish inmates.^9^ However, upon learning that she was a gynecologist, Mengele ordered her to use her specialty in the women’s camp.^10^ With no beds, instruments, or medication, Perl says that she “treated patients with my voice, telling them beautiful stories, telling them that one day we would have birthdays again, that one day we would sing again.”^9^

Perl’s greatest agony was the managing of pregnant women. She recalled: “Dr. Mengele told me that it was my duty to report every pregnant woman to him.”^9^

The discovered women were all exterminated. Upon realizing the fate of these women, Perl decided that there would never again be a pregnant woman in Auschwitz. The decision cost her dearly, but she realized that if she had not ended the pregnancies, both the mothers and their children would have faced certain death.^2^,^9^

Perl began to perform surgeries which before the war she had believed herself incapable of—abortions. In spite of her professional and religious beliefs as a doctor and an observant Jew, Dr. Perl began performing abortions on dirty floors with her bare, unwashed hands.^2^,^9^ Without any medical instruments or anesthesia, and often in the cramped and filthy bunks within the women’s barracks of Auschwitz, Perl ended the lives of countless fetuses, in hope that the mothers would survive and later, perhaps, be able to bear children. In some instances, the pregnancy was too far along to be able to perform an abortion. Perl would pierce the amniotic sac and manually dilate the mother’s cervix to induce labor. In these cases, the premature infants died, almost instantly. With the threat of a discovered pregnancy removed, the women were able to continue to work, gaining a temporary reprieve from their death sentences.

### Liberation

At the end of 1944, killing via the gas chambers ended. The crematorium was demolished, and the prisoners were taken on a forced death march. Perl, however, was transferred to a camp near Hamburg and then to Bergen-Belsen, a camp that she called “the supreme fulfillment of German sadism and bestiality.”^2^,^9^ On the very day that she was liberated by the British Army, Perl delivered a Jewish prisoner’s baby into the free world.

She wandered in Germany for months after the liberation, looking for her family. In 1947, after learning that all except her daughter had perished, Perl tried to end her life. Fortunately, she survived and eventually immigrated to New York.

### Perl’s Post-war Medical Contribution

Once in the US, Perl was initially suspected of war crimes—however, her testimony became critical in the conviction of at least one doctor in the Auschwitz trials and was markedly similar to the testimonies of other Auschwitz survivors.^10^

Perl began to speak about her experiences. It was after one such meeting that she met Eleanor Roosevelt, who prompted her to continue her life and medical service.^11^ With her help, Perl became a US citizen and opened a private obstetric practice in Manhattan. The majority of her patients were Holocaust survivors. She eventually became an infertility specialist at Mount Sinai Hospital.^11^ She would go on to deliver more than 3,000 babies.^9^,^11^,^12^ She additionally contributed to medical research and was the sole author or co-author of nine publications related to vaginal and urinary tract infections.^e.g.^^13^–^15^

At the end of her life, she joined her surviving daughter in Herzliya, Israel, where she lived until the age of 81.

## DR. ERNO VADASZ: PROTECTOR OF BABIES

In early 1945, when the tide of war was clearly turning against the Third Reich, several pregnant Jewish women managed to survive in the concentration camps, together with their newborns.^4^,^16^–^19^

A very special example was the “Pregnancy Unit” (*Schwanger Kommando*) in the Kaufering sub-camp of Dachau. Malnourished, exhausted, and low in weight, seven women with growing abdomens had not hid their secret. Surprisingly, they were not murdered. Instead, they were housed in a barrack and fed by a Jewish Kapo in charge of the kitchen. The Kapo recruited a Jewish obstetrician, a prisoner in the men’s camp, to perform the deliveries of the babies. The heroism of the mothers was complemented by the heroism of Dr. Erno Vadasz^19^ (personal communication from S. Magda to the authors, 2017) who is perhaps best remembered for these words, “too many Jewish children were killed, these must survive.”^4^,^20^

### Early Life and Work

**Figure 3:**
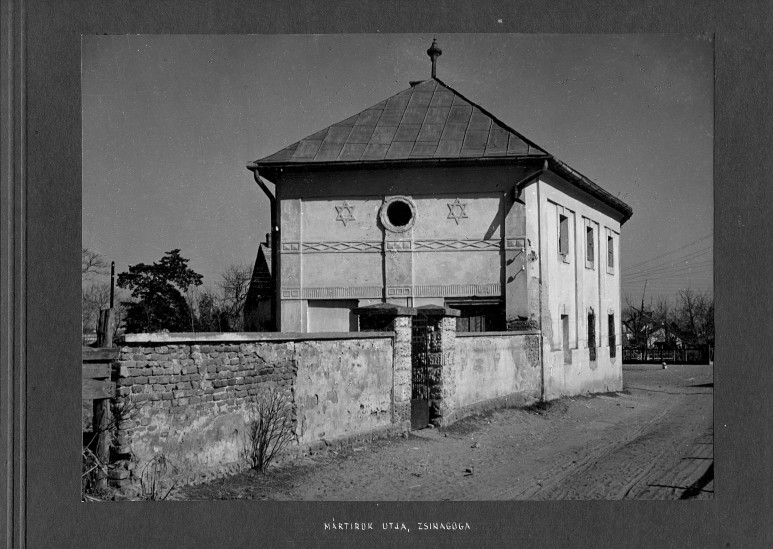
The Synagogue in the Hungarian Town of Nagykallo, Hungary Reproduced with permission from the Nagykallo Museum.

Erno Weisz was born in 1890 in the small Hungarian town of Nagykallo (Figure 3). The son of the local butcher, he completed high school in 1908. Weisz then changed his name to Vadasz, to help avoid exclusion from his studies due to the “numerus clausus” code restricting Jewish students. He excelled in his studies and continued his education in the Medical Faculty of the University of Budapest, graduating in 1913.

Soon after the start of his specialization, with the outbreak of World War I, Vadasz was conscripted into the Austro-Hungarian Army. After two months at the front, he was transferred for two years to a military hospital. Following his discharge from the Army, Vadasz completed his studies in obstetrics and gynecology. By 1930, he was well established and loved by the local population. He raised a family with his strictly orthodox wife and had two children.

### Deportation to Kaufering

In 1944, the family was deported to Auschwitz. Vadasz was subsequently transported to Kaufering, a sub-camp of Dachau. There, he became emaciated and so weak that he needed a prop in order to stand.

In February 1945, Vadasz was called to the women’s “Pregnancy Unit” by the Kapo, David Witz. Although he needed help to stand, Vadasz demanded “soap, knife, hot water, towels,” as for any delivery.^4^,^18^–^20^ The mothers had been well fed before the deliveries, and within a few weeks Vadasz had successfully brought all seven babies into the world. This despite the fact that two of the deliveries were complicated. One mother was bleeding with a retained placenta that had to be manually extracted. During another delivery, the infant was lying in a breech position; the labor was prolonged and almost unsuccessful—with a leg presentation. As the delivery progressed, another mother promised to care for the baby if the mother died. Despite his own weakness, Vadasz applied all of his medical skills, and both mother and child survived.^20^,^21^

Following the deliveries, one of the mothers developed pneumonia. Vadasz sat next to her as she lay, semi-conscious for two weeks, and cared for both mother and child, sharing his own food until she recovered. Vadasz also managed to save a young girl from the crematorium, whom he recognized from his own town. The last baby he delivered was born one day after demolition of the crematorium, on April 29, 1945.^21^

**Figure 4:**
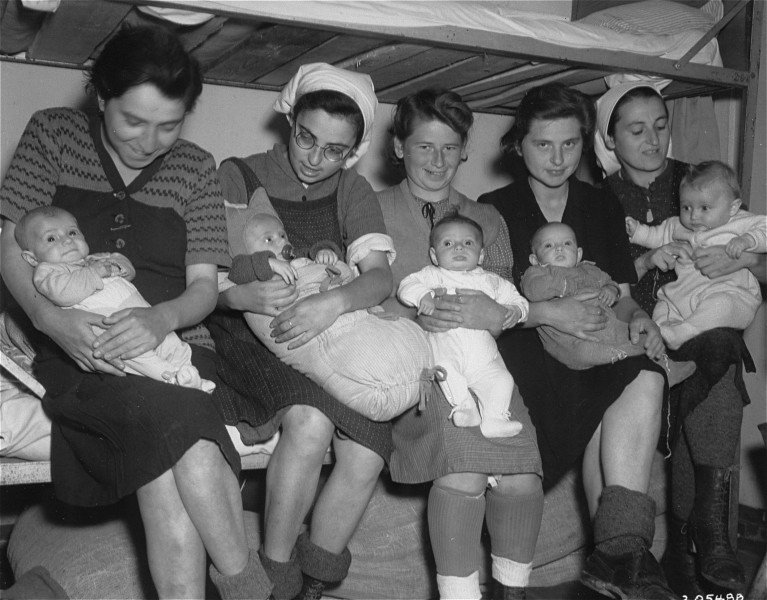
Group portrait of five Hungarian Jewish mothers and their infants in a Dachau sub-camp in Germany Pictured from left to right are: Ibolya Kovacs with her daughter Agnes; Suri Hirsch with her son Yossi; Eva Schwartz with her daughter Maria; Magda Fenyvesi with her daughter Judit; and Boeszi Legmann with her son Gyuri. Not pictured are: Dora Loewy and her daughter Szuszi; and Miriam Schwarcz Rosenthal and her son Laci (Leslie). Photo courtesy of United States Holocaust Memorial Museum, courtesy of National Archives and Records Administration, College Park; Public Domain.

In all, seven mothers were found by the liberating American Army, in satisfactory condition (see Figure 4).

### After the War

Following the liberation, Vadasz learned that his entire family had been murdered. Vadasz was never rewarded or recognized for the lives of the babies he delivered (most English sources only refer to a Hungarian Jewish gynecologist who helped the mothers and delivered the babies^17^). Anecdotal history, provided by the local Memorial Museum in Nagykallo (curator Mrs. Geza Harsanyi), suggests that Vadasz received false papers to leave the camp, accompanied by a supervising nurse. This is probable, perhaps as a reward for having successfully treated the commandant’s wife, but remains unverifiable, as it is unlikely that it would have been recorded in the camp diary (personal communication from S. Magda to the authors, 2017).

**Figure 5:**
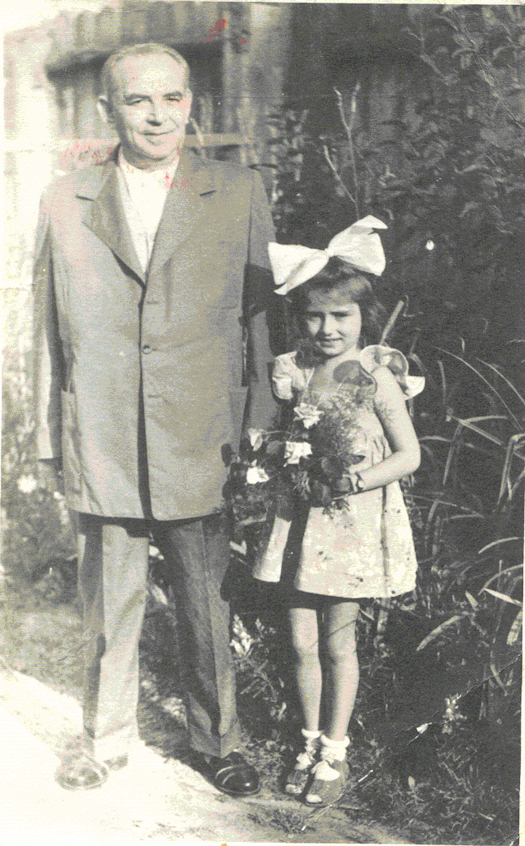
Post-war Photo of Dr. Erno Vadasz with the Daughter of a Patient Reproduced with permission from the Nagykallo Museum.

Vadasz returned to his hometown and restarted his practice; he was deeply loved and appreciated by the local residents (Figure 5). He married the supervising nurse from Dachau but refused to have children for fear of what might happen to them.^5^ He looked after the poor in the town and only charged what his patients could afford for his services.

He passed away in 1957 from prostatic carcinoma. A small, unpretentious room in the local historical museum was established in memory of this dedicated Jewish physician.

All but one of the mothers survived to their eighties and all the children were alive as of 2018. The story of their survival, as well as the question of how such a sudden humanitarian event was allowed to happen in the vicious camp, is puzzling. It must have been a calculated move by the otherwise brutal commandant facing eventual future legal proceedings.^17^,^20^,^21^

## CONCLUSION

Remembering persecuted physicians is a particularly sensitive chapter in Jewish history. This topic has been referred to repeatedly in Israeli medical journals, as historical records were discovered showing that such physicians were often considered to be heroes and innovators, despite the injustice and persecution that they suffered. Certainly, Vadasz and Perl were not the only Jewish physicians or gynecologists forced to work within the concentration camps. However, most of the others were murdered with their patients, and their stories will never be known. Hence, remembering Vadasz and Perl honors them as well.

The Jewish sage, Hillel, said: “If I am not for myself, who will be for me? But if I am only for myself, who am I? If not now, when?”^22^ Someone who makes the choice to act in the present for the benefit of others, while risking themselves, is considered a hero. Both Perl and Vadasz must be viewed as heroes in light of the risks they faced. Although some sought to accuse Perl of collaboration, she vehemently rejected such accusations and gave convicting testimony of what she saw and experienced in the camps.^6^–^8^,^10^ Both physicians faced challenges to the medical code of ethics and to various Jewish religious writings.^5^

Despite the different dilemmas and decisions of both physicians, there are striking similarities: (1) Both acted at risk to their own lives; (2) Both could have chosen different courses of action; (3) Both recognized the ultimate value of life; and (4) Both paid a price for their decisions. Ultimately, these are universal issues that may affect any physician anywhere, depending on his or her situation. Historic context merely contributes to the dilemmas each one faces—in their cases a dark moment in history.

Perl risked her life in many ways—if caught she would have been sent to the chambers; yet she took lives to save lives. Vadasz, on the other hand, risked his own life—health-wise—to help mothers give birth. Weak and sick himself, he delivered the babies under difficult, to say the least, circumstances, and even nursed one mother back to health. Any one of those events could have killed him in his weakened condition.

Both physicians remained human within an inhuman world, and their commitment to save lives remained despite the loss of their own families. Both physicians, in opposite ways, helped in the birth of future generations. The ethical dilemmas faced by them, their courage, and their example must be remembered.

Medical decision-making often involves multiple parameters, including the personal motivation of the physician, input from external influencers such as family members or hospital or national policy, as well as the need of the patient. It is not impossible to conceive a situation where a recommendation of great potential benefit to the patient could represent a significant personal risk to the physician, such as loss of prestige, loss of a position by going against a recommended policy, or alienation of family members. Perl and Vadasz faced the ultimate risk—that of their very lives. However, they considered the risk worthwhile in light of the potential benefit to their patients. May this standard and their example serve as an inspiration for future generations of physicians.
